# Exosomal HMGB1 Promoted Cancer Malignancy

**DOI:** 10.3390/cancers13040877

**Published:** 2021-02-19

**Authors:** Jiaan-Der Wang, Ya-Yu Wang, Shih-Yi Lin, Cheng-Yi Chang, Jian-Ri Li, Shi-Wei Huang, Wen-Ying Chen, Su-Lan Liao, Chun-Jung Chen

**Affiliations:** 1 Children’s Medical Center, Taichung Veterans General Hospital, Taichung City 407, Taiwan; jiaander@vghtc.gov.tw; 2 Department of Industrial Engineering and Enterprise Information, Tunghai University, Taichung City 407, Taiwan; 3 Department of Family Medicine, Taichung Veterans General Hospital, Taichung City 407, Taiwan; yywang@vghtc.gov.tw; 4 Institute of Clinical Medicine, National Yang Ming University, Taipei City 112, Taiwan; sylin@vghtc.gov.tw; 5 Center for Geriatrics and Gerontology, Taichung Veterans General Hospital, Taichung City 407, Taiwan; 6 Department of Surgery, Feng Yuan Hospital, Taichung City 420, Taiwan; changcy@fyh.mohw.gov.tw; 7 Division of Urology, Taichung Veterans General Hospital, Taichung City 407, Taiwan; roger@vghtc.gov.tw; 8 Translational Cell Therapy Center, China Medical University Hospital, Taichung City 404, Taiwan; t32207@mail.cmuh.org.tw; 9 Institute of New Drug Development, China Medical University, Taichung City 404, Taiwan; 10 Department of Veterinary Medicine, National Chung Hsing University, Taichung City 402, Taiwan; wychen@dragon.nchu.edu.tw; 11 Department of Medical Research, Taichung Veterans General Hospital, Taichung City 407, Taiwan; slliao@vghtc.gov.tw; 12 Department of Medical Laboratory Science and Biotechnology, China Medical University, Taichung City 404, Taiwan

**Keywords:** antiplatelet, exosome, HMGB1, malignancy

## Abstract

**Simple Summary:**

In addition to their role in hemostasis and thrombosis, platelets have been implicated in cancer malignancy and thrombocytosis in cancer patients and have been associated with an adverse prognosis. These phenomena indicate that antiplatelet drugs may be useful as an anticancer therapy. Using K562-differentiated megakaryocytes and murine platelets, conditioned medium and exosomes obtained from megakaryocytes and platelets contained high-mobility group box 1 (HMGB1) and promoted cancer cell survival, as well as protected cancer cells from doxorubicin cytotoxicity. Data of tumor-bearing mice established by Lewis lung carcinoma (LLC) cells and C57BL/6 mice revealed that antiplatelet drug dipyridamole and exosome release inhibitor GW4869 mitigated tumor growth and ameliorated concurrent alterations in blood circulation and tumor tissues, as well as platelet infiltration in tumor tissues. Therefore, exosomes and exosomal HMGB1 appear to have roles in platelet-driven cancer malignancy and represent targets of antiplatelet drugs in anticancer treatment.

**Abstract:**

Reciprocal crosstalk between platelets and malignancies underscores the potential of antiplatelet therapy in cancer treatment. In this study, we found that human chronic myeloid leukemia K562 cell-differentiated megakaryocytes and murine platelets produced bioactive substances and these are released into the extracellular space, partly in their exosomal form. High-mobility group box 1 (HMGB1) is a type of exosomal cargo, and the antiplatelet drugs aspirin and dipyridamole interfered with its incorporation into the exosomes. Those released substances and exosomes, along with exogenous HMGB1, promoted cancer cell survival and protected cells from doxorubicin cytotoxicity. In a tumor-bearing model established using murine Lewis lung carcinoma (LLC) cells and C57BL/6 mice, the tumor suppressive effect of dipyridamole correlated well with decreased circulating white blood cells, soluble P-selectin, TGF-β1 (Transforming Growth Factor-β1), exosomes, and exosomal HMGB1, as well as tumor platelet infiltration. Exosome release inhibitor GW4869 exhibited suppressive effects as well. The suppressive effect of dipyridamole on cancer cell survival was paralleled by a reduction of HMGB1/receptor for advanced glycation end-products axis, and proliferation- and migration-related β-catenin, Yes-associated protein 1, Runt-related transcription factor 2, and TGF- β1/Smad signals. Therefore, exosomes and exosomal HMGB1 appear to have roles in platelet-driven cancer malignancy and represent targets of antiplatelet drugs in anticancer treatment.

## 1. Introduction

Bloodstream platelets are small anucleated cell fragments released from bone marrow megakaryocytes. Besides their key roles in hemostasis and thrombosis, multifunctional platelets are also involved in numerous biological activities, including malignancy. Thrombocytosis is an adverse prognostic factor of malignancies. Cancer patients often exhibit thrombocytosis and platelet activation, which improve after surgical removal of the tumor mass [1,2,3]. Conversely, clinical and experimental findings indicate that defective platelet release, reduction of platelet count, and inactivation of platelets relieve tumor burden [4,5,6]. Despite advances in tumor treatments, therapeutic outcomes of malignancies are still unsatisfactory. Therefore, a comprehensive understanding of the crosstalk between platelets and malignancies may facilitate the development of molecular-based alternative strategies for combating malignancy.

Platelets arise from the cytoplasm of megakaryocyte, a unique hematological polyploid cell. Inside the platelets, bioactive molecules and enzymes are stored in lysosomes, dense granules, and α-granules, while plasma membrane of platelets is equipped with multiple glycoproteins. Once activated by engagement with specific ligands, degranulation and release of stored molecules of platelets occur. Through membrane-bound glycoproteins and released bioactive molecules, platelets contribute to and participate in tumor growth, angiogenesis, dissemination, metastasis, immune escape, and resistance [7,8]. Additionally, microparticles and exosomes are emerging vehicles of platelets that coordinate both local and distant tumor–host crosstalk [9,10].

Reciprocal crosstalk between platelets and malignancies underscores the clinical importance of antiplatelet therapy in cancer patient treatment. Clinical and experimental studies demonstrate that antiplatelet therapy reduces the incidence of malignancies and malignant progression by targeting platelet receptors, interfering with platelet granule release, or inhibiting platelet-specific enzymes. Among the antiplatelet drugs, aspirin is the most common candidate, while dipyridamole is attracting growing interest [11,12,13,14,15].

High-mobility group box 1 (HMGB1), a nuclear chromatin-binding protein, displays tumor-promoting effects in malignancies. Aspirin inhibits platelet–tumor cell interaction and tumor metastasis by targeting tumor HMGB1 expression, release, and action [8,9,16]. The preventive role of low-dose aspirin against atherosclerosis complications is attributable to the reduction of platelet aggregation by inhibition of HMGB1 release from platelets and megakaryocytes [17]. The aforementioned findings suggest that HMGB1 could be an action target of antiplatelet drugs involving the disruption of reciprocal interaction between platelets and cancer cells. Previously, we reported the anticancer effects of aspirin through apoptosis, growth arrest, metabolic inhibition, and platelet cargo reduction, such as soluble P-selectin (sP-selectin) and TGF- β1 [18,19,20]. To continue and extend our research concentrating on reciprocal crosstalk between platelets and cancer cells, as well as anticancer potential of antiplatelet drugs, human chronic myeloid leukemia K562 cell-differentiated megakaryocytes, human bladder cancer T24 cells, murine platelets, murine Lewis lung carcinoma (LLC) cells, and C57BL/6-LLC tumor-bearing mice models were established and subjected to antiplatelet drug treatment. The reciprocal crosstalk between platelets and cancer cells as well as the potential involvement of HMGB1 were further investigated using dipyridamole.

## 2. Materials and Methods

### 2.1. Cell Culture

Murine LLC (#60050), human bladder carcinoma T24 (#60062), and human chronic myeloid leukemia K562 (#60007) cells were purchased from the Bioresource Collection and Research Center (Hsinchu, Taiwan). T24 cells were maintained in McCoy’s 5a medium, while LLC and K562 cells were cultured in RPMI-1640 medium containing 1% nonessential amino acids and 10% fetal bovine serum (FBS) in a 5% CO_2_ incubator at 37 °C.

### 2.2. Morphological Assay

K562 cells were treated with phorbol 12-myristate 13-acetate (PMA) (0 and 10 nM) for 4 days. Cytospins were obtained by centrifugation at 200 rpm, spread onto glass slides, and stained with Wright–Giema dye. Morphological observation and lobulation of the nucleus were analyzed under a light microscope.

### 2.3. Caspase 3 Activity Assay

To measure caspase 3 activity, T24 cells were seeded onto 6-well plates prior to treatments. Cell lysates were harvested and equal amounts of proteins were reacted with substrate buffer for the measurement of caspase 3 activity according to the instructions of the Caspase Fluorometric Assay Kit (BioVision, Mountain View, CA, USA). Relative caspase 3 activity was expressed as fluorescence changes measured with a fluorometer (E_x_ 380 nm and E_m_ 460 nm) per amounts of protein.

### 2.4. Colony Formation Assay

To measure long-term cell growth, LLC cells (500 cells per well) were plated onto 6-well plates in RPMI-1640 medium supplemented with 5% FBS. One day after seeding, the cells were treated with various concentrations of dipyridamole (0–25 µM) for 6 days. To visualize cell colonies, the cells were stained with crystal violet.

### 2.5. Motility Assay

To perform wound-healing assay, LLC cells were seeded onto 6-well plates, and growth was maintained until confluence was achieved. A wound was created in the confluent cell monolayer by scratching with a tip of a 200-µL micropipette maintained in RPMI-1640 medium with 0.5% FBS. Photomicrographs were taken immediately after the scraping and before treatment and 24 h later. A 24-well Transwell permeable chamber with 8 µM pore size (BD Falcon Cell Culture insert, BD Biosciences, San Jose, CA, USA) was used for the measurement of cell migration. LLC cells (2 × 10^4^) in 200 µL RPMI-1640 medium containing 2% FBS were added to the top of the Transwell inserts with or without treatment, and 600 µL RPMI-1640 medium containing 10% FBS was put into the lower chambers of Transwell for 24 h. The transmigratory cells in the lower surfaces of the Transwell inserts were stained with Giema, and the cell numbers in six random fields per inserts were counted for comparison.

### 2.6. Platelet Preparation

Platelet preparation and tumor growth were performed in C57BL/6 mice. The study protocols were reviewed and approved by the Animal Experimental Committee of Taichung Veterans General Hospital (IACUC approval code: La1081613, IACUC approval date: 10 January 2019). The procedures of platelet preparation were performed according to a previously described method with modifications [17]. Whole blood from male C57BL/6 mice (10 weeks old, *n* = 20) were collected in ACD buffer (citric acid, 39 mM; sodium citrate, 75 mM; dextrose, 135 mM) via cardiac puncture under anesthesia with 2% isoflurane. The samples were centrifuged at 400× *g* for 40 min. The supernatants were collected, mixed with prostaglandin E1 (0.25 µM), and centrifuged at 1250× *g* for 15 min. Cell pellets were resuspended with ACD buffer containing prostaglandin E1 (0.25 µM) and centrifuged again. The final pellets were resuspended with phosphate-buffered saline (PBS) or RPMI-1640 medium for further experiments.

### 2.7. Cell Viability Assay

To measure cell viability, LLC and T24 cells were seeded onto 96-well plates prior to treatments. An assay kit (CellTiter 96 AQ_ueous_ Non-Radioactive Cell Proliferation Assay Kit, Promega, Madison, WI, USA) containing a novel tetrazolium compound (3-(4,5-dimethylthiazol-2-yl)-5-(3-carboxymethoxyphenyl)-2-(4-sulfophenyl)-2H-tetrazolium, inner salt; MTS) was utilized to measure cell viability. Its bioreduction by cells into a formazan was measured by a spectrophotometer at 490 nm. For the analysis of conditioned medium (CM), cultured media of K562/PMA-differentiated megakaryocytes and platelets were collected and centrifuged at 2000 rpm for 10 min. The supernatants were collected and mixed with an equal volume of fresh medium, termed CM.

### 2.8. Flow Cytometric Assay

To determine cell apoptosis, T24 cells were collected, resuspended with PBS, and fixed with 70% ethanol. Then, the cells were incubated with propidium iodide (PI)/RNase A mixture at 4 °C for 30 min. The cells were analyzed by flowcytometry. To analyze the expression of surface markers, K562/PMA-differentiated megakaryocytes, platelets, and exosomes were incubated with fluorescein isothiocyanate (FITC) isotype (#553964, BD Biosciences, San Jose, CA, USA), phycoerythrin (PE) isotype (#12-4714-82, Thermo Fisher Scientific, Rockford, IL, USA), PE-labeled anti-cluster of differentiation 41 (CD41) (#133906, Biolegend, Biolegend, San Diego, CA, USA), PE-labeled anti-CD9 (#555372, BD Biosciences, San Jose, CA, USA), and FITC-labeled anti-CD61 (#104306, Biolegend, San Diego, CA, USA) at room temperature for 40 min. The levels of cell surface marker proteins were measured using the FACSCalibur and analyzed by Cell Quest Pro software (BD Biosciences, San Jose, CA, USA). The amounts of CD41 on cell surface were expressed as the genomic mean fluorescence intensity.

### 2.9. Syngeneic Tumor Model Study

Under anesthesia with isoflurane, LLC cells (LLL, 1 × 10^5^ cells in 100 µL of serum-free DMEM) and equal volumes of normal saline (Saline) were inoculated subcutaneously into the right flanks of the male C57BL/6 mice (8 weeks old). Three days later, LLC cell-implanted (LLC) and saline (Saline) mice were divided into three subgroups (*n* = 8 per subgroup), respectively, and began to receive daily doses of dipyridamole (10 mg/kg, po), GW4869 (2.5 mg/kg, ip), or saline administration. After 18 days, the mice were euthanized by isoflurane and CO_2_ inhalation and sacrificed. Their tumors were resected for measurement and analyses. Tumor volume was calculated according to the following formula: V = (L × W^2^)/2 (L = length; W = width) [20].

### 2.10. Blood Sample Collection and Analyses

At the end of the studies, mice were euthanized with isoflurane and CO_2_ inhalation and blood samples were withdrawn from the left femoral artery. The numbers of white blood cells (WBC) and platelets were measured by complete blood count. The plasma levels of sP-selectin (R&D Systems, Minneapolis, MN, USA) and TGF-β1 (R&D Systems, Minneapolis, MN, USA) were determined using enzyme-linked immunosorbent assay (ELISA) kits, following the procedures provided by the manufacturers.

### 2.11. Exosome Isolation

At the end of the studies, mice were euthanized with isoflurane and CO_2_ inhalation and blood samples were withdrawn from the left femoral artery. Exosomes were isolated from blood samples using Invitrogen Total Exosome Isolation Kit (from plasma) (Invitrogen 4484450, Thermo Fisher Scientific, Waltham, MA, USA). For the isolation of exosomes from cell cultures, Invitrogen Total Exosome Isolation Reagent (from cell culture media) (Invitrogen 4478359, Thermo Fisher Scientific, Waltham, MA, USA) was used. All of the reagents were provided by the kits, and procedures adhered to the manufacturers’ instructions. The use of exosome isolation kits and protocols was referred to relevant studies [21,22].

### 2.12. Western Blot

Proteins were extracted from cells and resected tumor tissues using tissue protein reagents (T-PER, Thermo Fisher Scientific, Rockford, IL, USA) and subjected to standardized SDS-PAGE. Proteins recognized by specific antibodies included, CD41 (sc-53711), CD42a (sc-166420), CD42b (sc-59052), CD61 (sc-7312), Cyclin D1 (sc-450), β-catenin (sc-59737), phosphorylated Smad2/3 (sc-11769), glyceraldehyde-3-phosphate dehydrogenase (GAPDH, sc-32233), Yes-associated protein 1 (YAP1) (sc-376830), Runt-related transcription factor 2 (Runx2) (sc-390351), toll-like receptor 2 (TLR2) (sc-10739), TLR4 (sc-10741), receptor for advanced glycation end-products (RAGE) (sc-74473), CD9 (sc-18869), heat shock Protein 70 (HSP70) (sc-66048) (Santa Cruz Biotechnology, Santa Cruz, CA, USA), and HMGB1 (ab77302, Abcam, Cambridge, MA, USA). The blots were then incubated with the appropriate horseradish peroxidase-labeled IgG, visualized using enhanced chemiluminescence (ECL) Western blotting reagents, and quantified by ImageJ software (Version 1.52u, National Institute of Health, Bethesda, MD, USA).

### 2.13. Statistical Analysis

All values are expressed as means ± standard deviations. Statistical comparisons were analyzed using one-way or two-way analysis of variance. Tukey or Dunnett post hoc tests were used for multiple comparisons. Differences were considered statistically significant at a *p* value less than 0.05.

## 3. Results

### 3.1. Megakaryocyte-Conditioned Medium Promoted T24 Cell Survival

**Figure 1:**
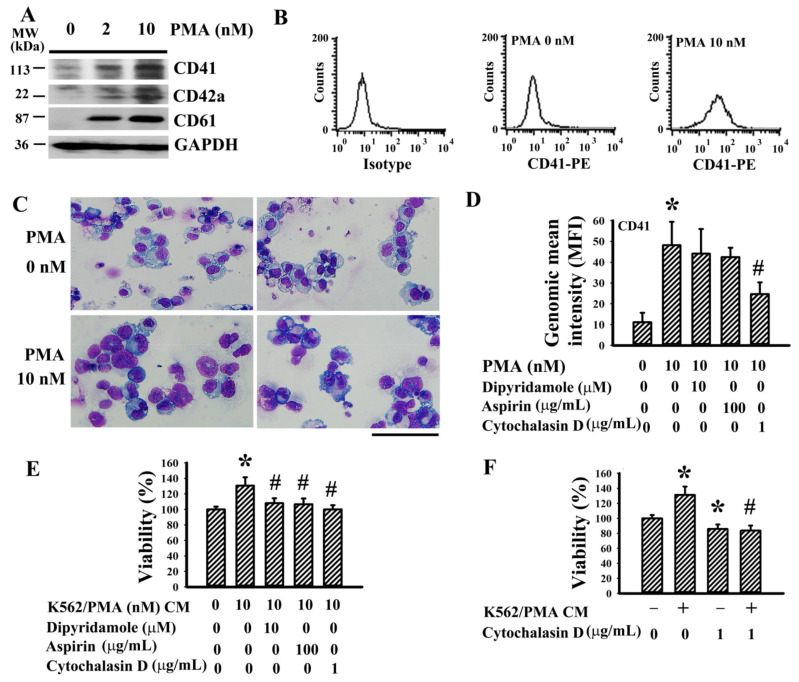
Conditioned medium of megakaryocytes promoted T24 cell survival. K562 cells were treated with various concentrations of phorbol 12-myristate 13-acetate (PMA) (0–10 nM) for 4 days. (**A**) Proteins were extracted and subjected to Western blot analysis with indicated antibodies. Representative blots of four independent experiments are shown. The Western blots have been shown in Figure S1. (**B**) Cells were harvested and subjected to flowcytometric analysis for the measurement of surface presentation of CD41. (**C**) Cells were pelleted by centrifugation, spread onto glass slides, and stained with Wright–Giemsa dye. Scale bar: 300 µm. K562 cells were treated with PMA (0 and 10 nM) in the absence or presence of dipyridamole (10 µM), aspirin (100 µg/mL), or cytochalasin D (1 µg/mL) for 4 days. (**D**) Cells were harvested and subjected to flowcytometric analysis for the measurement of surface presentation of CD41. Bar graphs show relative genomic mean intensity of CD41-PE fluorescence. (**E**) The cultured media were harvested and centrifuged, and the resultant supernatants were named conditioned medium (CM). Equal amounts of CM and fresh medium were mixed and added to T24 cells (96-well) for 48 h. Cell viability was measured by the 3-(4,5-dimethylthiazol-2-yl)-5-(3-carboxymethoxyphenyl)-2-(4-sulfophenyl)-2H-tetrazolium (MTS) reduction assay. (**F**) Equal amounts of CM obtained from PMA treatments (0 and 10 nM) and fresh medium were mixed and added to T24 cells (96-well) in the absence or presence of cytochalasin D (1 µg/mL) for 48 h. Cell viability was measured by the MTS reduction assay. * *p* < 0.05 vs. untreated group and # *p* < 0.05 vs. PMA (10 nM) group or K562/PMA (10 nM) CM group, *n* = 4.

K562 cell line is a common model of megakaryocytic differentiation and pro-platelet formation stimulated by PMA [23,24]. Upon treatment with PMA, K562 cells increased protein expression of CD41, CD42a, and CD61 (Figure 1A), elevated cell surface presentation of CD41 (Figure 1B), and formed larger cells with multi-lobulation of the nucleus (Figure 1C), demonstrating successful megakaryocytic differentiation. The antiplatelet drugs dipyridamole and aspirin had little effect on PMA-elevated CD41 cell surface presentation, while actin cytoskeleton disruptor cytochalasin D decreased it (Figure 1D). Conditioned medium of K562/PMA-differentiated megakaryocytes promoted T24 cell survival, and the effects were attenuated by the presence of dipyridamole, aspirin, or cytochalasin D during megakaryocytic differentiation (Figure 1E). Intriguingly, the pro-survival effects were also attenuated by the presence of cytochalasin D in T24 cells upon exposure to conditioned medium (Figure 1F). The findings suggest the potential release of pro-survival molecules by K562/PMA-differentiated megakaryocytes, and the recipient cells likely have an intact actin cytoskeleton to respond to.

### 3.2. Exosomes and HMGB1 Contributed to the Pro-Survival Effects of Conditioned Medium

**Figure 2:**
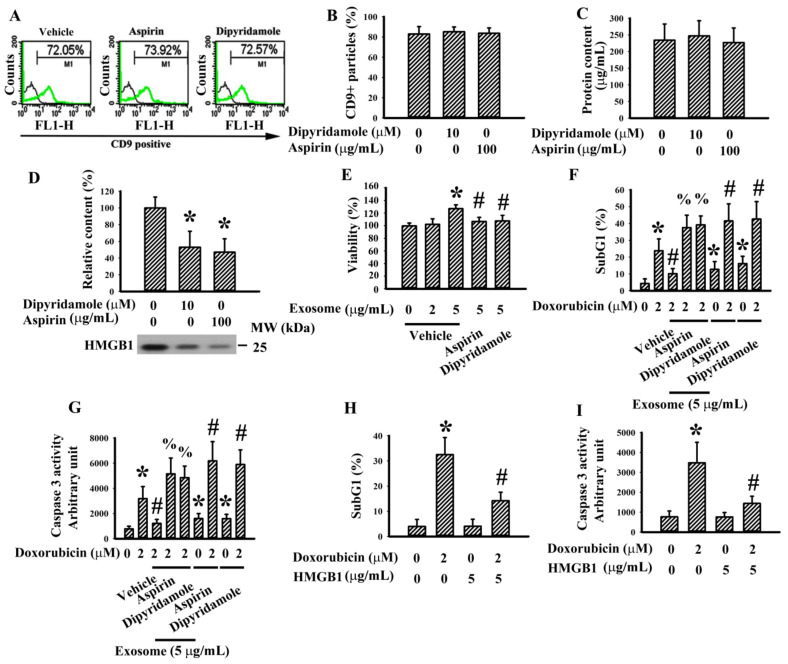
Megakaryocytes produced pro-survival exosomes. K562 cells were treated with PMA (10 nM) in the absence or presence of dipyridamole (10 µM) or aspirin (100 µg/mL) for 4 days. Equal amounts of cultured media were subjected to exosome isolation. (**A**,**B**) The obtained exosomes were subjected to flowcytometric analysis for the measurement of CD9 positive particles. (**C**) The obtained exosomes were subjected to the measurement of protein content. (**D**) The obtained exosomes were subjected to Western blot analysis with indicated antibodies. Representative blots of four independent experiments are shown. (**E**) Various concentrations of exosomes based on protein contents were added to T24 cells (96-well) for 48 h. Cell viability was measured by the MTS reduction assay. T24 cells were treated with doxorubicin (0 and 2 µM) in the absence or presence of the obtained exosomes (5 µg/mL), dipyridamole (10 µM), or aspirin (100 µg/mL) for 48 h. (**F**) Cells were harvested and subjected to flowcytometric analysis for the measurement of SubG1 population. (**G**) Proteins were extracted and subjected to enzymatic assay for the measurement of caspase 3 activity. T24 cells were treated with doxorubicin (0 and 2 µM) in the absence or presence of high-mobility group box 1 (HMGB1) (5 µg/mL) for 48 h. (**H**) Cells were harvested and subjected to flowcytometric analysis for the measurement of SubG1 population. (**I**) Proteins were extracted and subjected to enzymatic assay for the measurement of caspase 3 activity. * *p* < 0.05 vs. untreated group, # *p* < 0.05 vs. exosome (vehicle, 5 µg/mL) or doxorubicin (2 µM), and % *p* < 0.05 vs. doxorubicin (2 µM)/exosome (vehicle, 5 µg/mL), *n* = 4.

**Figure 3:**
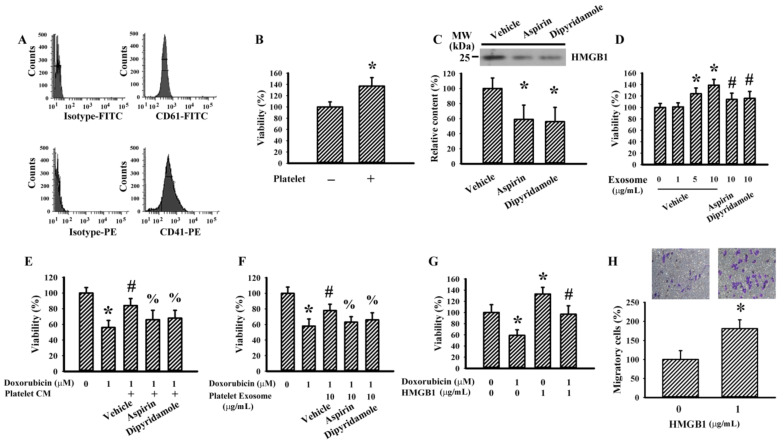
Conditioned medium and exosomes of platelets promoted Lewis lung carcinoma (LLC) cell survival. Blood was withdrawn from C57BL/6 mice and subjected to platelet isolation. (**A**) The isolated platelets were subjected to flowcytometric analysis for the measurement of CD41 and CD61 positivity. (**B**) Platelets and medium alone were added onto the Transwell inserts, and LLC cells were grown at the lower wells of Transwell apparatus for 24 h. Cell viability of LLC cells was measured by the MTS reduction assay. Platelets were treated with vehicle, dipyridamole (10 µM), or aspirin (100 µg/mL) for 24 h. Equal amounts of cultured media were subjected to exosome isolation. (**C**) The obtained exosomes were subjected to Western blot analysis with indicated antibodies. Representative blots of four independent experiments are shown. (**D**) Various concentrations of exosomes based on protein contents were added to LLC cells (96-well) for 24 h. Cell viability was measured by the MTS reduction assay. (**E**) The cultured media were harvested and centrifuged, and the resultant supernatants were named platelet-conditioned medium (CM). Equal amounts of CM and fresh medium were mixed and added to LLC cells (96-well) in the absence or presence of doxorubicin (0.5 µM) for 24 h. Cell viability was measured by the MTS reduction assay. (**F**) LLC cells were treated with doxorubicin (0 and 1 µM) in the absence or presence of the obtained exosomes (10 µg/mL) for 24 h. Cell viability was measured by the MTS reduction assay. (**G**) LLC cells were treated with doxorubicin (0 and 1 µM) in the absence or presence of HMGB1 (1 µg/mL) for 24 h. Cell viability was measured by the MTS reduction assay. (**H**) Cell migration of LLC cells in the absence or presence of HMGB1 (1 µg/mL) was evaluated using Transwell migration assay for 24 h. * *p* < 0.05 vs. untreated group, # *p* < 0.05 vs. exosome (vehicle, 10 µg/mL)/doxorubicin, and % *p* < 0.05 vs. doxorubicin (CM or Exosome), *n* = 4.

Exosomes and HMGB1 are commonly bioactive molecules of platelets and megakaryocytes [9,17]. K562/PMA-differentiated megakaryocytes produced and released CD9^+^ exosomes (Figure 2A) and the levels of released exosomes were not altered by the presence of dipyridamole and aspirin during megakaryocytic differentiation (Figure 2B). Exosomes isolated from equal amounts of K562/PMA-differentiated megakaryocyte-conditioned medium contained a relatively constant level of total proteins independent of the presence of dipyridamole or aspirin (Figure 2C). There was HMGB1 protein present in the exosomes and its content decreased in the presence of dipyridamole and aspirin (Figure 2D). An addition of K562/PMA-differentiated megakaryocyte-derived exosomes promoted T24 cell survival. However, the effects decreased in the presence of dipyridamole and aspirin during megakaryocytic differentiation (Figure 2E). Anticancer drug doxorubicin generated SubG1 cell population (Figure 2F) and increased caspase 3 activity (Figure 2G) in T24 cells. K562/PMA-differentiated megakaryocyte-derived exosomes protected against doxorubicin cytotoxicity, and the effects were reversed by the presence of dipyridamole and aspirin during megakaryocytic differentiation (Figure 2F and Figure 3G). Furthermore, the presence of dipyridamole and aspirin in T24 cells augmented doxorubicin cytotoxicity (Figure 2F,G). Additionally, an addition of HMGB1 directly attenuated doxorubicin cytotoxicity in T24 cells (Figure 2H,I). That is, K562/PMA-differentiated megakaryocytes produced functional exosomes containing HMGB1, which promoted T24 cell survival and protected T24 cells from doxorubicin cytotoxicity. Dipyridamole and aspirin could interfere with these effects and could be mimicked by HMGB1.

### 3.3. Murine Platelets Promoted LLC Cell Survival

To further explore the biological implications of reciprocal interaction between human megakaryocytes and T24 cells, the following studies were performed on murine platelets and LLC cells. Murine platelets were prepared from C57BL/6 mice and confirmed by the positivity of surface presentation of CD 41 and CD61 (Figure 3A). Platelets separated by Transwell inserts promoted LLC cell survival (Figure 3B). Platelet-derived exosomes contained HMGB1 (Figure 3C) and promoted LLC cell survival (Figure 3D). The presence of dipyridamole and aspirin decreased exosomal HMGB1 content (Figure 3C) and pro-survival effects (Figure 3D). Platelet-derived conditioned medium (Figure 3E) and exosomes (Figure 3F) protected LLC cells from doxorubicin cytotoxicity, and the effects were attenuated by the presence of dipyridamole and aspirin. Exogenous HMGB1 promoted LLC cell survival (Figure 3G), protected cells from doxorubicin cytotoxicity (Figure 3G), and increased LLC cell migration (Figure 3H). The findings further reveal the pro-survival effects of platelet-derived conditioned medium and exosomes toward LLC cells, and exosomal HMGB1 may have an active role.

### 3.4. Dipyridamole Decreased LLC Cell Viability and Migration

**Figure 4:**
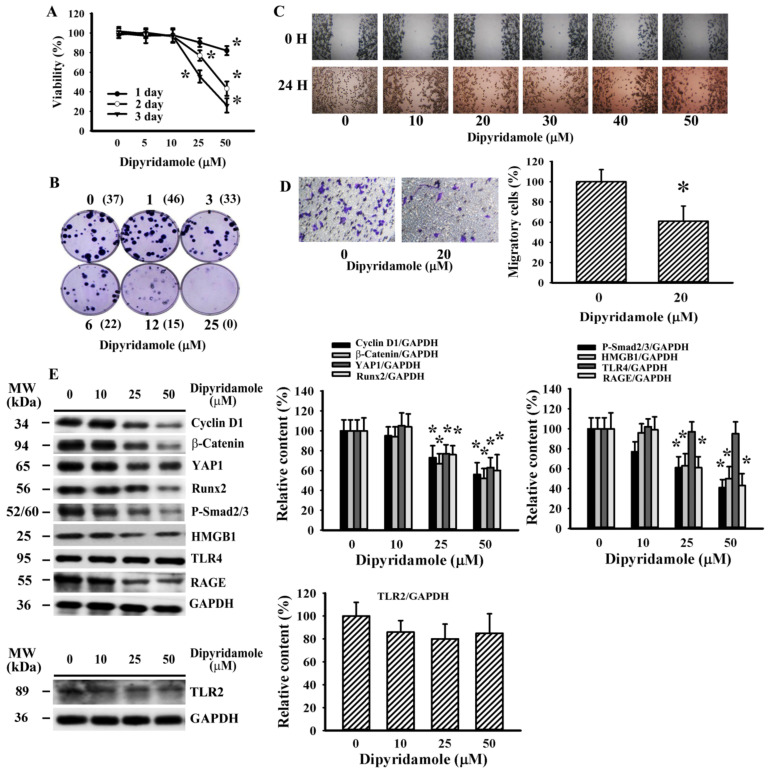
Dipyridamole mitigated LLC cell proliferation. (**A**) LLC cells were treated with various concentrations of dipyridamole (0–50 µM) over time. Cell viability was evaluated by MTS reduction assay. (**B**) LLC cells were treated with various concentrations of dipyridamole (0–25 µM) for 6 days. Cell colonies were fixed and stained with crystal violet. Colony numbers are shown in parentheses. (**C**) Cell movement was evaluated by a wound-healing assay in confluent LLC cells with various concentrations of dipyridamole (0–50 µM) for 24 h. Representative photomicrographs are shown. (**D**) LLC cells were seeded onto Transwell inserts in the presence of dipyridamole (0 and 20 µM) and subjected to Transwell migration assay for 24 h. The lower chambers were filled with RPMI-1640 medium containing 10% FBS. (**E**) LLC cells were treated with various concentrations of dipyridamole (0–50 µM) for 16 h. Proteins were extracted and subjected to Western blot with indicated antibodies. Representative blots of four independent experiments, and quantitative data are shown. * *p* < 0.05 vs. untreated group, *n* = 4.

In addition to its use as an antiplatelet drug, dipyridamole also displays cancer cell-killing effects [25]. There was a decline of LLC cell viability (Figure 4A) and long-term cell growth (Figure 4B) upon dipyridamole treatment. Low concentrations of dipyridamole caused decreased gap closure in a wound-healing assay (Figure 4C) and a reduction of transmigration in a Transwell migration assay (Figure 4D). Dipyridamole had an inhibitory effect on HMGB1 and RAGE protein expression, with the exception of TLR2 and TLR4 (Figure 4E). Additionally, protein levels of cell proliferation- and migration-associated cyclin D1, β-catenin, YAP1, Runx2, and phosphorylated Smad2/3 decreased upon dipyridamole treatment (Figure 4E). The findings indicate a direct effect of dipyridamole on LLC cell proliferation and migration.

### 3.5. Dipyridamole Mitigated Tumor Growth in Tumor-Bearing Mice

**Figure 5:**
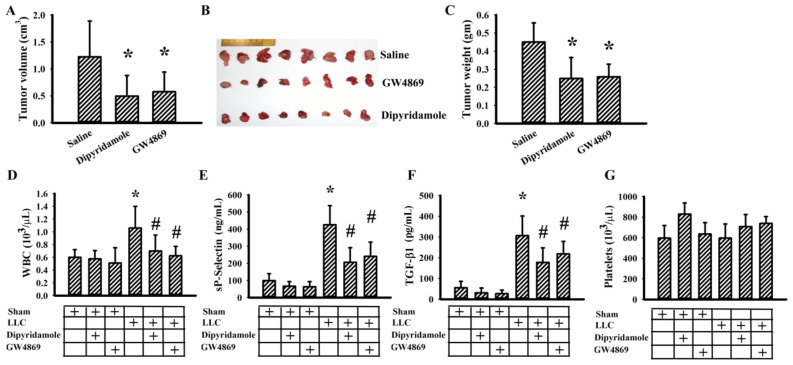
Dipyridamole and GW4869 mitigated tumor growth. LLC cells or saline vehicle were implanted into C57BL/6 mice and allowed to grow for 3 weeks. Three days after implantation, dipyridamole (10 mg/kg) and GW4869 (2.5 mg/kg) were administrated daily up until the end of the experiment. The tumor volume (**A**), resected tumor tissues (**B**), and tumor mass (**C**) are shown. The total white blood cells (WBC) (**D**), sP-selectin (**E**), TGF-β1 (**F**), and platelets (**G**) in blood samples were determined. * *p* < 0.05 vs. saline or sham untreated group and # *p* < 0.05 vs. LLC untreated group, *n* = 8.

**Figure 6:**
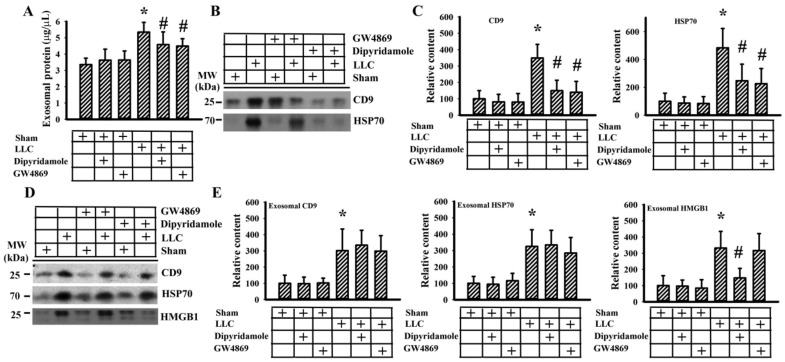
Dipyridamole and GW4869 decreased exosome content. LLC cells or saline vehicle were implanted into C57BL/6 mice and were allowed to grow for 3 weeks. Three days after implantation, dipyridamole (10 mg/kg) and GW4869 (2.5 mg/kg) were administrated daily up until the end of the experiment. Equal amounts of blood samples were subjected to exosome isolation. Protein contents of the isolated exosomes were measured (**A**). Proteins were extracted from the isolated exosomes and subjected to Western blot with indicated antibodies. Representative blots (**B**) and quantitative data (**C**) are shown. Proteins were extracted from equal amounts of isolated exosomes and subjected to Western blot with indicated antibodies. Representative blots (**D**) and quantitative data (**E**) are shown. * *p* < 0.05 vs. sham untreated group and # *p* < 0.05 vs. LLC untreated group, *n* = 8.

**Figure 7:**
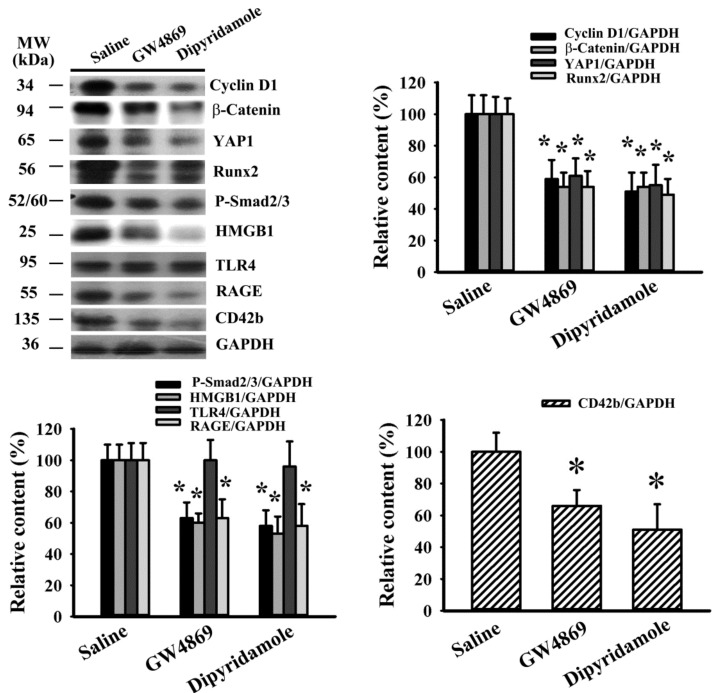
Dipyridamole and GW4869 decreased signaling molecule expression in tumor tissues. LLC cells or saline vehicle were implanted into C57BL/6 mice and allowed to grow for 3 weeks. Three days after implantation, dipyridamole (10 mg/kg) and GW4869 (2.5 mg/kg) were administrated daily up until the end of the experiment. Proteins were extracted from the resected tumor tissues and subjected to Western blot with indicated antibodies. Representative blots and quantitative data are shown. * *p* < 0.05 vs. sham untreated group, *n* = 8.

Previously, we reported that aspirin mitigated tumor growth in vivo [20]. To further extend in vitro findings with respect to antiplatelet drugs and exosomes, the effects of dipyridamole and GW4869, an inhibitor of exosome release [26], were explored in C57BL/6-LLC tumor-bearing mice. Dipyridamole and GW4869 mitigated tumor growth (Figure 5A–C). Tumor-bearing mice showed an increased number of WBC (Figure 5D), circulating sP-selectin (Figure 5E), and TGF-β1 (Figure 5F), without alteration in platelet number (Figure 5G). Tumor-bearing mice increased exosomal protein content in blood circulation (Figure 6A) and exosomal cargo proteins CD9 and HSP70 (Figure 6B,C) [27]. The elevations were alleviated by dipyridamole and GW4869 (Figure 5 and Figure 6A–C). To further examine the composition of released exosomes, protein contents were measured in an equal number of exosomes. Exosomal CD9, HSP70, and HMGB1 were not significantly altered by GW4869, nor were exosomal CD9 and HSP70 by dipyridamole. Instead, there was a reduction of exosomal HMGB1 induced by dipyridamole (Figure 6D,E). In examining the dissected tumor tissues, both dipyridamole and GW4869 decreased protein contents in cyclin D1, β-catenin, YAP1, Runx2, phosphorylated Smad2/3, HMGB1, RAGE, and CD42b, except the TLR4 (Figure 7). That is, the in vivo tumor growth was accompanied by increased platelet activation, exosome release, exosomal HMGB1, and tumor platelet infiltration, and dipyridamole and GW4869 displayed ameliorating effects.

## 4. Discussion

The involvement of platelets physically and functionally in cancer growth, metastasis, and malignant progression is well recognized. The association of lower mortality rate with clinical use of aspirin and other antiplatelet drugs underscores the potential utility of antiplatelet drugs in cancer treatment [11]. Platelet-released bioactive substances and platelet-tumor cell aggregates contribute to the pro-proliferative, pro-angiogenic, and pro-metastatic effects of platelets [7,8,9,10]. Using K562-differentiated megakaryocytes and murine platelets, conditioned medium and exosomes obtained from megakaryocytes and platelets contained HMGB1 and promoted cancer cell survival, as well as protected cancer cells from doxorubicin cytotoxicity. The aforementioned pro-survival effects were attenuated by dipyridamole and aspirin and were mimicked by exogenous HMGB1. Furthermore, dipyridamole caused a reduction of cell viability, long-term cell growth, and migration in LLC cells, accompanied by decreased expression of the HMGB1/RAGE axis and proliferation- and migration-associated molecules. Tumor-bearing mice, established by LLC cells and C57BL/6 mice, showed increased levels of circulating WBC, sP-selectin, and TGF-β1, and produced an elevated level of exosome in the bloodstream. Antiplatelet drug dipyridamole and exosome release inhibitor GW4869 mitigated tumor growth and ameliorated concurrent alterations in blood circulation and tumor tissues, as well as platelet infiltration in tumor tissues. Moreover, dipyridamole displayed an additional effect in reducing exosomal HMGB1 content. Therefore, exosomes and exosomal HMGB1 appear to have roles in platelet-driven cancer malignancy and represent targets of antiplatelet drugs in anticancer treatment.

Platelet glycoprotein receptors such as GPVI, GPIb-IX-V (CD42/CD42a/CD42d), GPIbα (CD42b), GPIbβ (CD42c), GPIIb-IIIa (CD41/CD61), P-selectin (CD62P), and G protein-coupled receptors have substantial roles in platelet adhesion, activation, and aggregation. There are many bioactive substances stored in the secretory organelles of platelets released at the basal level or at an accelerated level upon platelet activation. Cytokines, chemokines, thrombin, ADP, serotonin, thromboxane A2, vascular endothelial growth factor (VEGF), platelet-derived growth factor (PDGF), and TGF-β1 are ubiquitous components of platelets and have been implicated in cancer malignancy and inflammation. Additionally, platelet exosomes represent an alternative vehicle for the delivery of bioactive substances in coordinating local and distant effects, and HMGB1 is an emerging exosomal cargo [7,8,9,17,28]. In syngeneic mice, tumor growth was accompanied by elevated circulating WBC, sP-selectin, TGF-β1, exosomes, and exosomal HMGB1, along with tumor HMGB1 and CD42b. In parallel, K562/PMA-differentiated megakaryocytes and murine platelets produced soluble substances and exosomes containing HMGB1. Like exogenous HMGB1, their conditioned medium and exosomes presented cancer cell pro-survival and protective effects. Although the sources of exosomes, exosomal HMGB1, and TGF-β1 production in the bloodstream vary, our findings suggest that megakaryocytes and platelets are likely origins. However, due to the lack of any direct evidence, the contribution of other sources cannot be ignored.

Numerous antiplatelet drugs have been developed by targeting platelet receptors, granules, or enzymes. Aspirin is a pharmacological inhibitor of cyclooxygenase, and dipyridamole is a phosphodiesterase inhibitor [11,12,13,14,15]. The production of soluble substances with cancer cell pro-survival effects by megakaryocytes and platelets was hampered by dipyridamole and aspirin. Intriguingly, dipyridamole and aspirin significantly suppressed HMGB1 cargo protein levels of megakaryocytes- and platelets-derived exosomes without altering total levels of exosomes. Furthermore, disruption of actin cytoskeleton in recipient cells abolished the pro-survival effects of conditioned medium. In human platelets, aspirin blocks thrombin- and collagen-induced increases in exosome cargo levels of chemokines and HMGB1, without altering total exosome secretion [9]. Antiplatelet clopidogrel, an ADP receptor blocker, alters exosome cargo components in cancer cells [29]. The cAMP/PKA signaling impairs cytoplasmic translocation and release of HMGB1 [30]. Exosome, a type of extracellular vesicle with membranes characterized by specific exosomal markers, such as CD9, CD63, CD81, and HSP70, is involved in intercellular communications [27]. Platelet exosomal cargoes include cytokines, chemokines, coagulation factors, growth factors, HMGB1, and RNAs. Since exosomes are crucial mediators of platelets in coordinating cancer malignancy and inflammation [9,31], our findings support the results of relevant studies and further highlight the fact that exosomes are targets of the biological activities of antiplatelet drugs. Unfortunately, the mechanisms underlying the interference of specific incorporation of platelet exosomal cargoes by antiplatelet drugs have yet to be identified. Since dipyridamole is a phosphodiesterase inhibitor, the cAMP/PKA signaling might be candidate for its effect on K562 cells and platelets. Therefore, the effects of cAMP analogues and/or effects of PKA inhibitors on dipyridamole’s actions should be investigated in continuous studies.

Besides their influence on platelets, antiplatelet drugs also display direct effects on cancer cells. Aspirin is reported to increase cancer cell chemotherapeutic responses by downregulation of ATP binding cassette (ABC) transporters [32]. Dipyridamole impairs autophagic flux, inhibits cell proliferation, induces apoptosis, antagonizes TGF-β1 activity, and decreases in vivo tumor growth [25,33,34]. It also synergizes with agents to induce cancer cell apoptosis [35,36]. Phosphodiesterase inhibitors increase the efficacy of anticancer drugs by promoting endocytosis-mediated cellular drug uptake [37]. We have demonstrated that aspirin induced endoplasmic reticulum stress-increased Noxa upregulation, restored ABT-737 apoptosis, and impaired glucose and glutamine metabolism in cancer cells [18,19,20]. In this study, aspirin and dipyridamole caused cell apoptosis and sensitized cells to doxorubicin-induced apoptosis. Inhibition of cancer cell survival, migration, and in vivo tumor growth by dipyridamole was further demonstrated in LLC cells and a tumor-bearing model in C57BL/6 mice. Regarding the underlying mechanism of the anticancer effects of dipyridamole, our findings further suggest that the tumor effect of the HMGB1/RAGE axis is a crucial target.

The pleiotropic effects of HMGB1 depend on its subcellular localization, nucleus, cytoplasm, or extracellular space. Cytoplasmic HMGB1 has a role in autophagy through the HMGB1/Beclin complex, while extracellular HMGB1 regulates cellular activities via TLR2, TLR4, or RAGE membrane receptors [38,39,40]. Platelet HMGB1 has been implicated in thrombosis, inflammation, and platelet activation involving TLR4/RAGE [39,41]. Tumor HMGB1 and TLR4/RAGE receptors promote cell proliferation, survival, migration, epithelial-to-mesenchymal transition, and apoptosis resistance involving interaction with β-catenin, Runx2, YAP1, and TGF-β1/Smad [38,42,43,44,45,46]. However, HMGB1 released from dying tumor cells may stimulate bone marrow-derived tumor-infiltrating myeloid dendritic cells through HMGB1/TLR2 signaling [40]. In an in vitro cell model or in vivo tumor tissues, dipyridamole treatment resulted in a decline in the levels of HMGB1, RAGE, β-catenin, Runx2, YAP1, phosphorylated Smad2/3, and cyclin D1, but not TLR2 and TLR4. Similarly, in in vivo tumor tissues, GW4869 caused a reduction of those molecules. The effects of GW4869 in tumor-bearing mice were paralleled by a reduction of circulating WBC, sP-selectin, TGF-β1, exosomes, and exosomal HMGB1. Thus, blood-borne, platelet-derived exosomal HMGB1 putatively exerts pro-malignant effects through the tumor HMGB1/RAGE axis. However, it should be noted that alternative mechanisms beyond exosomal HMGB1 underlying local tumor growth may exist owing to the presence of platelets within tumor tissues. Platelet-tumor cell aggregates, tumor origin HMGB1, and unidentified molecules and mechanisms are likely to be active in the reciprocal crosstalk between platelets and tumor cells.

## 5. Conclusions

Herein, we found that megakaryocytes and platelets produced bioactive substances that were released into the extracellular space, partly through exosomal forms. HMGB1 is a type of exosomal cargo, and aspirin and dipyridamole interfered with its incorporation into the exosomes. Those released substances and exosomes, along with exogenous HMGB1, promoted cancer cell survival and protected cells from doxorubicin cytotoxicity. The cancer cell survival suppressive effect of dipyridamole was paralleled by a reduction of the HMGB1/RAGE axis, and proliferation- and migration-related β-catenin, YAP1, Runx2, TGFβ1/Smad, and cyclin D1 in in vitro and in vivo tumor growth models. The anticancer effects of antiplatelet drugs are multifactorial and vary. Further research is warranted to explore the roles of exosomes and exosomal HMGB1 in platelet-driven cancer malignancy and to investigate the anticancer targets of antiplatelet drugs.

## Data Availability

Not applicable.

## Supplementary Materials

The following are available online at https://www.mdpi.com/2072-6694/13/4/877/s1, Figure S1: The western blots figures.
