# Supplementary material for: Exosomal HMGB1 Promoted Cancer Malignancy
Source: Cancers (Basel). 2021 Feb 19;13(4):877. doi: 10.3390/cancers13040877 (PMC7921955; doi:10.3390/cancers13040877)
Supplement: Supplementary file 1 [file cancers-13-00877-s001.pdf]

cancers

Supplementary Materials: Exosomal HMGB1 Promoted Cancer Malignancy

Jiaan-Der Wang, Ya-Yu Wang, Shih-Yi Lin, Cheng-Yi Chang, Jian-Ri Li, Shi-Wei Huang, Wen-Ying Chen, Su-Lan Liao and Chun-Jung Chen

Figure 1A

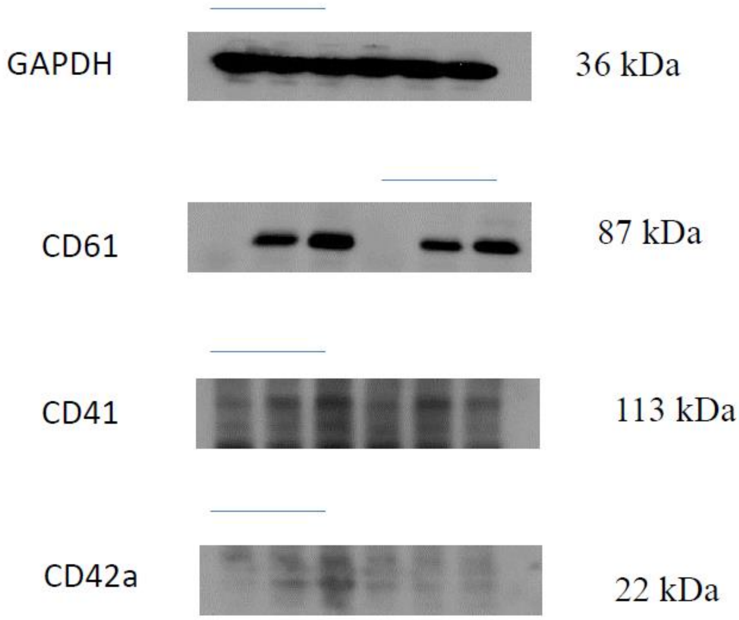

Figure 2D

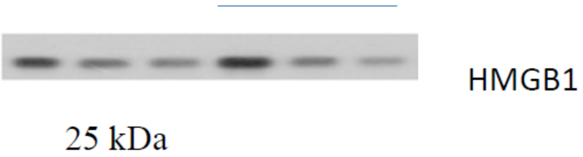

8459.0000 6014.0000
3158.0000
7124.0000 3875.0000
4963.0000
6695.0000 5519.0000
5201.0000
9275.0000 4012.0000
3856.0000

Figure 3C

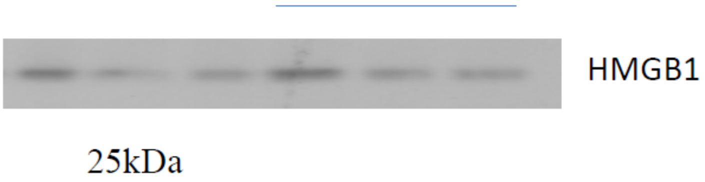

6857.0000 2856.0000
4702.0000
5214.0000 2014.0000
2415.0000
6025.0000 4836.0000
2096.0000
4869.0000 3702.0000
3725.0000

Figure 4E

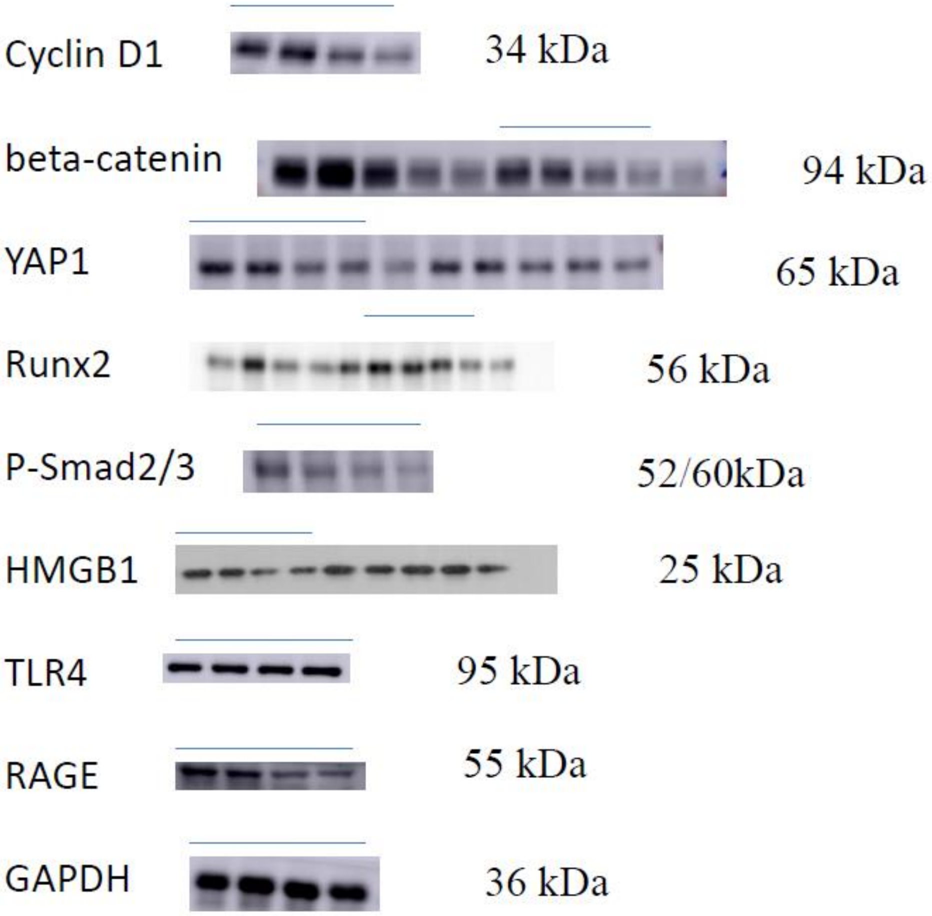

1.2300	1.0900	0.9700	0.8000
1.0200	0.9700	0.7600	0.5900
1.1600	1.2400	0.8100	0.7900
1.3500	1.1600	1.0300	0.6700

Cyclin D1

beta-catenin

YAP1

Runx2

Ratio/GAPDH

1.1900	0.9600	0.9400	0.4300
1.2700	1.1100	0.7200	0.7000
1.0300	1.1700	0.6400	0.5900
1.1000	1.0900	0.8500	0.7400
1.3600	1.2800	0.8100	0.6200

1.0200	1.0900	0.6700	0.6300
0.8400	1.1800	0.8500	0.7400
0.9600	1.0600	0.7400	0.4900
1.1300	0.8600	0.6600	0.5700

0.9400	0.7900	0.7400	0.6700
1.0800	1.0700	0.6500	0.4700
0.8500	1.0200	0.5900	0.5500
0.7600	0.8600	0.8000	0.6800

P-Smad2/3

HMGB1

TLR4

RAGE

Ratio/GAPDH

1.0600	0.7000	0.6200	0.4000
0.9800	0.7300	0.5400	0.3800
0.8600	0.6000	0.4300	0.4900
0.9000	0.6700	0.5100	0.2900

0.8900	0.6900	0.5700	0.3200
0.6700	0.7200	0.4600	0.5100
0.8200	0.8500	0.6100	0.4600
0.7900	0.7900	0.4200	0.3700

0.9500	0.9000	0.8600	0.8200
0.7600	0.7500	0.7400	0.9400
0.8400	0.8800	0.8200	0.6700
0.9200	0.9300	0.9400	0.8100

1.0600	0.8000	0.6700	0.4200
1.1400	1.1100	0.5800	0.5600
0.9800	1.0200	0.4600	0.4900
0.8200	0.8700	0.5100	0.2700

Figure 6B

CD9

25 kDa

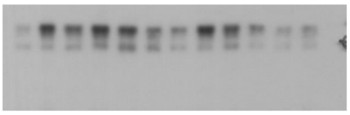

1100.0000	2400.0000	2700.0000	6800.0000	3800.0000
1600.0000	1600.0000	1600.0000	7200.0000	1900.0000
2800.0000	700.0000	3400.0000	9400.0000	2700.0000
3600.0000	500.0000	2800.0000	10600.0000	2300.0000
3700.0000	1100.0000	2100.0000	8400.0000	1500.0000
2100.0000	3500.0000	700.0000	6200.0000	3400.0000
1700.0000	1600.0000	600.0000	8200.0000	5500.0000
1900.0000	2700.0000	400.0000	6700.0000	2600.0000

HSP70

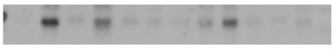

70 kDa

1600.0000	1800.0000	2100.0000	6700.0000	2600.0000	2700.0000	3500.0000
700.0000	600.0000	300.0000	12400.0000	3700.0000	3900.0000	1600.0000
3700.0000	2700.0000	1700.0000	8400.0000	4100.0000	6800.0000	2400.0000
1600.0000	2400.0000	1600.0000	5900.0000	5400.0000	2800.0000	800.0000
2400.0000	1100.0000	900.0000	8400.0000	1600.0000	5100.0000	600.0000
900.0000	700.0000	1700.0000	8700.0000	2100.0000	4600.0000	1900.0000
500.0000	900.0000	400.0000	9900.0000	4900.0000	4800.0000	5200.0000
1700.0000	1700.0000	2900.0000	11200.0000	3400.0000	2600.0000	4900.0000

Figure 6D

25 kDa CD9

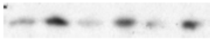

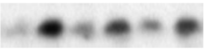

70 kDa

HSP70

500.0000	2400.0000	2100.0000	9500.0000	6700.0000	6600.0000
1700.0000	1100.0000	1600.0000	7400.0000	8200.0000	5700.0000
2600.0000	1900.0000	3400.0000	5600.0000	7300.0000	8200.0000
3400.0000	800.0000	2500.0000	3200.0000	6800.0000	4900.0000
1800.0000	2700.0000	1700.0000	4100.0000	3900.0000	7300.0000
2400.0000	3400.0000	1600.0000	2900.0000	5300.0000	9200.0000
1600.0000	1600.0000	2200.0000	8500.0000	4800.0000	8400.0000
3300.0000	2200.0000	1800.0000	7700.0000	7600.0000	3500.0000

HMGB1

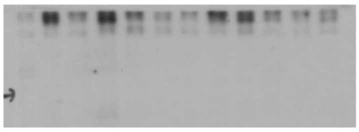

25 kDa

1200.0000	2700.0000	1600.0000	6200.0000	7200.0000
1600.0000	1300.0000	3400.0000	8400.0000	3400.0000
3400.0000	2500.0000	900.0000	7100.0000	5600.0000
2700.0000	1900.0000	2500.0000	3600.0000	8200.0000
1600.0000	700.0000	1600.0000	5900.0000	4600.0000
2400.0000	800.0000	2700.0000	8400.0000	3800.0000
1600.0000	2900.0000	3300.0000	5300.0000	7200.0000
2300.0000	1600.0000	1600.0000	6600.0000	9100.0000

1600.0000	1300.0000	3200.0000	3500.0000	3200.0000	4600.0000	6300.0000
3500.0000	1600.0000	1500.0000	4600.0000	1600.0000	3900.0000	4500.0000
4100.0000	700.0000	1900.0000	7600.0000	700.0000	8200.0000	7200.0000
1300.0000	2200.0000	2400.0000	6900.0000	2700.0000	6600.0000	4900.0000
2500.0000	1800.0000	2600.0000	8400.0000	4300.0000	7600.0000	6300.0000
1700.0000	1600.0000	1700.0000	7700.0000	3300.0000	5200.0000	5200.0000
900.0000	2400.0000	600.0000	8300.0000	7500.0000	3600.0000	4900.0000
700.0000	2100.0000	300.0000	9200.0000	6200.0000	4100.0000	7100.0000

Figure 7

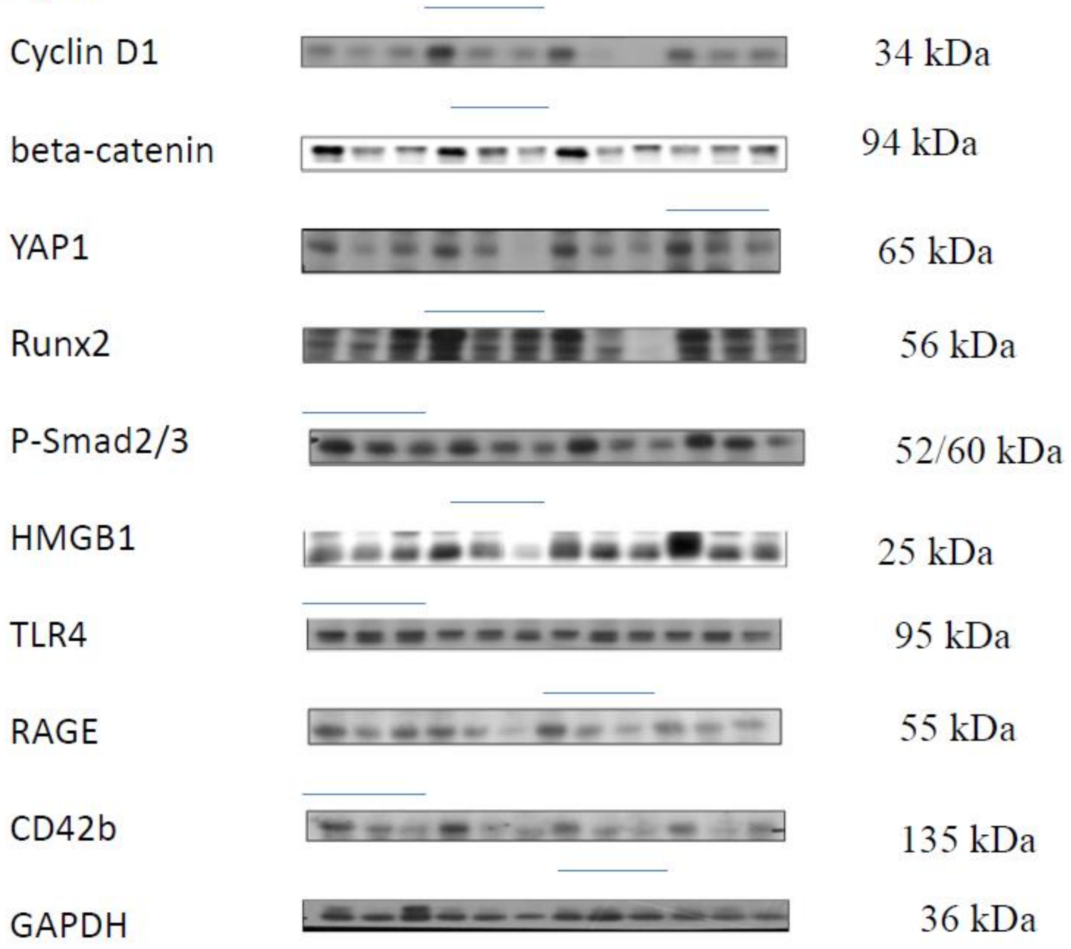

Cyclin D1

beta-catenin

YAP1

Runx2

Ratio/GAPDH

1.2900	0.7200	0.7600
1.1600	0.4900	0.6200
1.0800	0.6600	0.5100
1.1000	0.5700	0.4200
0.9500	0.8400	0.5300
0.8900	0.6500	0.6900
1.1100	0.7100	0.7200
1.0600	0.6300	0.5900

1.1700	0.6800	0.6900
1.0500	0.7200	0.7000
1.3000	0.5400	0.6500
1.2900	0.6100	0.8400
1.4500	0.8200	0.5100
1.2500	0.6500	0.7300
1.0300	0.7500	0.6900
1.1600	0.8000	0.7500

1.1300	0.6000	0.3700
1.0500	0.4900	0.4900
0.9400	0.7600	0.5100
0.8600	0.5200	0.6600
0.9300	0.6400	0.6700
0.9600	0.6300	0.5900
1.0800	0.5800	0.6400
1.0300	0.7100	0.3900

1.3700	0.8400	0.7000
1.2800	0.6200	0.5900
1.1900	0.9100	0.6700
1.3800	0.7200	0.8500
1.5700	0.6000	0.4900
1.2300	0.8500	0.6800
1.2700	0.7900	0.7100
1.1500	0.9300	0.6900

	1.3800	0.6600	0.6100	1.3900	0.7100	0.6400
P-Smad2/3	1.1500	0.6400	0.5700	1.2500	0.6900	0.5400
	1.2600	0.7400	0.7900	1.1900	0.7700	0.4900
HMGB1	1.0700	0.9100	0.8500	1.2300	0.8400	0.7600
	1.1900	0.8500	0.6900	1.0500	0.6500	0.8000
TLR4	0.0900	0.8600	0.5900	1.0900	0.8500	0.6200
	1.1800	0.7200	0.6700	1.2000	0.6700	0.5800
RAGE	1.1100	0.6900	0.8400	1.1300	0.7200	0.7600

Ratio/GAPDH

1.0900	1.0300	1.0800	1.0600	0.6200	0.7900
1.1200	1.1900	1.1600	1.1400	0.7800	0.6200
1.0100	0.9900	0.8500	0.9500	0.5900	0.4300
0.8700	0.8400	0.7600	0.8700	0.4500	0.5100
0.9200	0.9500	0.9400	0.9300	0.6700	0.5300
0.9000	0.8500	0.9300	0.9600	0.7500	0.6300
0.9700	0.7900	1.1600	1.0700	0.6400	0.5700
1.0900	1.0300	1.0500	1.1900	0.5300	0.4200

CD42b

Ratio/GAPDH

1.0600	0.5700	0.6200
1.1400	0.6900	0.7800
0.9500	0.7400	0.5100
0.9000	0.6200	0.4300
1.2100	0.8400	0.3500
0.9600	0.6800	0.3700
1.0500	0.7900	0.5200
1.1100	0.8000	0.6100

Figure S1. The western blots figures.
